# Type of calcineurin inhibitor and long-term outcomes following liver transplantation in patients with primary biliary cholangitis – an ELTR study

**DOI:** 10.1016/j.jhepr.2024.101100

**Published:** 2024-04-25

**Authors:** Maria C. van Hooff, Rozanne C. de Veer, Vincent Karam, Rene Adam, Pavel Taimr, Wojciech G. Polak, Hasina Pashtoun, Sarwa Darwish Murad, Christophe Corpechot, Darius Mirza, Michael Heneghan, Peter Lodge, Gabriel C. Oniscu, Douglas Thorburn, Michael Allison, Herold J. Metselaar, Caroline M. den Hoed, Adriaan J. van der Meer, for the European Liver and Intestine Transplant Association (ELITA), Darius Mirza, Michael Heneghan, Peter Lodge, Gabriel Oniscu, Douglas Thorburn, Michael Allison, Johann Pratschke, Derek Manas, William Bennet, Pal-Dag Line, Emir Hot, Krzysztof Zieniewicz, Bo Goran Ericzon, Jiri Fronek, Jurgen L. Klempnauer, Allan Rasmussen, Renato Romagnoli, Petr Nemec, Arno Nordin, Andreas Paul, Paolo De Simone, R.J. Porte, Gabriela Berlakovich, Daniel Cherqui, Jacques Pirenne, Etienne Sokal, Giorgio Rossi, Daniel Candinas, Philippe Bachellier, Oleg Rummo, Karim Boudjema, Anna Mrzljak, Olivier Soubrane, Herold Metselaar, Stefan Schneeberger, Francis Navarro, Thierry Berney, Christophe Duvoux, Michele Colledan, Luciano De Carlis, Olivier Boillot, Jean Hardwigsen, Francois Rene Pruvot, Bertrand Suc, Marco Vivarelli, Pierre Alain Clavien, Hauke Lang, Maciej Kosieradzki, Frederik Berrevoet, Bruno Heyd, Matteo Cescon, Laurence Chiche, Eberhard Kochs, Umberto Baccarani, Olivier Detry, Michael Bartels, Massimo Rossi, Olivier Soubrane, Olivier Scatton, Vasileios Papanikolaou, Ian Alwayn, Peter Schemmer, N. Senninger, Christian Ducerf, Fabrizio Di Benedetto, Giuseppe Tisone, Silvio Nadalin, Zoltan Mathe, Marija Ribnikar, Utz Settmacher, Thomas Becker, Nuno Silva, Jorge Daniel, Irinel Popescu, Valerio Lucidi, Wolf O. Bechstein, Thomas Decaens, Jean Gugenheim, Salvatore Gruttadauria, Frausto Zamboni, Murat Zeytunlu, Jorg C. Kalff, Toomas Vali, Yaman Tokat, Ernst Klar, Eberhard Kochs, Renato Romagnoli, Julius Janek, Murat Kilic, Krum Katzarov, Lutz Fisher, Emmanuel Buc, Marco Castagneto, Tarkan Unek, Lubomir Spassov, Dirk Stippel, Christiane Bruns, Hans Schlitt, Ephrem Salame, Piotr Kalicinski, Koray Acarli

**Affiliations:** 1 Department of Gastroenterology and Hepatology, Erasmus MC Transplant Institute, University Medical Center Rotterdam, The Netherlands; 2 European Society for Organ Transplantation, Amsterdam, The Netherlands; 3 European Liver Transplant Registry, Department of Hepatobiliary and Pancreatic Surgery and Liver Transplantation AP-HP Hôpital Paul Brousse, Université Paris-Saclay Villejuif, France; 4 Department of Hepatology and Gastroenterology, Institute for Clinical and Experimental Medicine, Prague, Czech Republic; 5 Department of Surgery, Division of HPB & Transplant Surgery, Erasmus MC Transplant Institute, University Medical Center Rotterdam, The Netherlands; 6 Reference Center for Inflammatory Biliary Diseases and Autoimmune Hepatitis, European Reference Network on Hepatological Diseases (ERN Rare-Liver), Saint-Antoine Hospital, Assistance Publique - Hôpitaux de Paris; Inserm UMR_S938, Saint-Antoine Research Center, Sorbonne University, Paris, France; 7 Department of HPB Surgery, Liver Unit, Queen Elizabeth Hospital, Birmingham, United Kingdom; 8 Institute of Liver Studies, King’s College Hospital, London, United Kingdom; 9 The Leeds Teaching Hospitals NHS Trust, Leeds, United Kingdom; 10 Edinburgh Transplant Centre, Royal Infirmary of Edinburgh, Edinburg, United Kingdom; 11 Division of Transplantation, CLINTEC, Karolinska Institutet, Stockholm, Sweden; 12 Department of Hepatology and Liver Transplantation, Royal Free Hospital, London, United Kingdom; 13 Liver Unit, Cambridge University Hospitals NHS Foundation Trust, Cambridge NIHR Biomedical Research Centre, Cambridge, United Kingdom

**Keywords:** Calcineurin inhibitors, Graft survival, Liver Transplantation, Primary Biliary Cholangitis, Survival

## Abstract

**Background & Aims:**

Tacrolimus has been associated with recurrence of primary biliary cholangitis (PBC) after liver transplantation (LT), which in turn may reduce survival. This study aimed to assess the association between the type of calcineurin inhibitor used and long-term outcomes following LT in patients with PBC.

**Methods:**

Survival analyses were used to assess the association between immunosuppressive drugs and graft or patient survival among adult patients with PBC in the European Liver Transplant Registry. Patients who received a donation after brain death graft between 1990 and 2021 with at least 1 year of event-free follow-up were included.

**Results:**

In total, 3,175 patients with PBC were followed for a median duration of 11.4 years (IQR 5.9–17.9) after LT. Tacrolimus (Tac) was registered in 2,056 (64.8%) and cyclosporin in 819 (25.8%) patients. Following adjustment for recipient age, recipient sex, donor age, and year of LT, Tac was not associated with higher risk of graft loss (adjusted hazard ratio [aHR] 1.07, 95% CI 0.92-1.25, *p =* 0.402) or death (aHR 1.06, 95% CI 0.90-1.24, *p =* 0.473) over cyclosporin. In this model, maintenance mycophenolate mofetil (MMF) was associated with a lower risk of graft loss (aHR 0.72, 95% CI 0.60-0.87, *p <*0.001) or death (aHR 0.72, 95% CI 0.59-0.87, *p <*0.001), while these risks were higher with use of steroids (aHR 1.31, 95% CI 1.13-1.52, *p <*0.001, and aHR 1.34, 95% CI 1.15-1.56, *p <*0.001, respectively).

**Conclusions:**

In this large LT registry, type of calcineurin inhibitor was not associated with long-term graft or recipient survival, providing reassurance regarding the use of Tac post LT in the population with PBC. Patients using MMF had a lower risk of graft loss and death, indicating that the threshold for combination treatment with Tac and MMF should be low.

**Impact and implications:**

This study investigated the association between immunosuppressive drugs and the long-term survival of patients with primary biliary cholangitis (PBC) following donation after brain death liver transplantation. While tacrolimus has previously been related to a higher risk of PBC recurrence, the type of calcineurin inhibitor was not related to graft or patient survival among patients transplanted for PBC in the European Liver Transplant Registry. Additionally, maintenance use of mycophenolate was linked to lower risks of graft loss and death, while these risks were higher with maintenance use of steroids. Our findings should provide reassurance for physicians regarding the continued use of Tac after liver transplantation in the population with PBC, and suggest potential benefit from combination therapy with mycophenolate.

## Highlights

• Long-term risks of graft loss or death were similar between patients with PBC on tacrolimus or cyclosporin after LT.
• This finding supports the continued use of tacrolimus in this population.
• Maintenance use of mycophenolate was associated with lower risks of graft loss and death.
• Maintenance use of steroids selected a group of patients with unfavourable long-term outcomes.

## Graphical abstract

**Figure:**
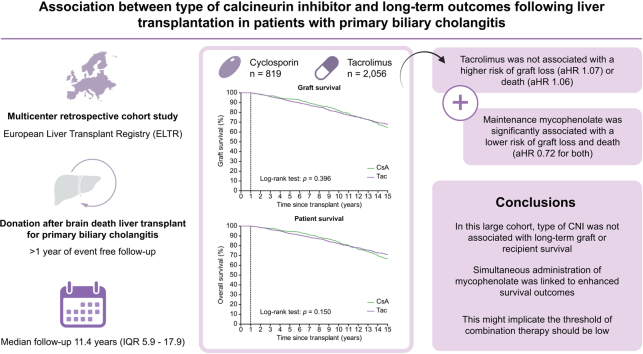

## Introduction

Primary biliary cholangitis (PBC) is a chronic and usually slowly progressive liver disease with autoimmune features, histologically characterized by destruction of the small intrahepatic bile ducts.^1^^,^^2^ The disease is mainly diagnosed in middle-aged women, based on elevated serum alkaline phosphatase levels and presence of anti-mitochondrial antibodies. The recommended standard treatment for PBC is ursodeoxycholic acid (UDCA), which delays histological disease progression and improves liver transplant (LT)-free survival.[3], [4], [5] Nonetheless, despite adequate UDCA therapy, a substantial proportion of patients still develop cirrhosis, at which stage they are at risk of liver failure and hepatocellular carcinoma (HCC).^6^ In particular, patients with an incomplete biochemical response to UDCA are at increased risk of hepatic fibrosis progression.^7^^,^^8^

At present, while treatment options are increasing with various nuclear receptor agonists, LT remains the only potentially curative treatment option for patients with PBC. LT is restricted to patients with decompensated liver disease, selected patients with HCC or those with poor quality of life due to unmanageable pruritus.[9], [10], [11] Even though UDCA treatment has become common practice, approximately 400 patients with PBC still undergo a transplantation in Europe and the United States each year.^12^^,^^13^ The outcome of LT in the population with PBC can be considered good. Within the European Liver Transplant Registry (ELTR), the 5-year graft and patient survival rates are 78% and 83%, respectively.^14^^,^^15^ This surpasses the 5-year graft and patient survival rates post LT in patient with other aetiologies of chronic liver disease such as viral hepatitis (65% and 74%), primary sclerosing cholangitis (72% and 82%), autoimmune hepatitis (73% and 79%), and alcohol-related cirrhosis (71% and 75%), or primary liver tumours (61% and 64%).^14^^,^^15^ Still, graft and patient survival in patients with PBC may be compromised, as PBC recurs in 17-46% of patients in the years following LT.^16^ Recurrence of PBC (rPBC) was recently shown to be associated with impaired graft and patient survival.^17^

In general, with respect to calcineurin inhibitors (CNIs), tacrolimus (Tac) is preferred over cyclosporin (CsA) following LT based on the lower rate of acute cellular rejection (ACR) and graft loss.^11^^,^^16^ Nevertheless, use of Tac in the setting of LT for PBC is a topic of ongoing debate as there have been some reports suggesting a higher rate of rPBC in those treated with Tac.[17], [18], [19] However, most of these cohort studies were limited by a relatively small number of included patients. In addition, prior efforts have not generally assessed the association between the type of CNI and graft loss or patient mortality as the most solid clinical endpoints. Accordingly, the primary aim of this study was to assess the association between the type of CNI and long-term graft and patient survival following LT for PBC within the large dataset of the ELTR. In parallel, we aimed to assess the association between other immunosuppressive drugs and these clinical outcomes after LT.

## Patients and methods

### Study population

This study was performed within the ELTR, which includes prospectively collected data on patients undergoing LT in nearly all European liver transplant units (174 centres in 32 countries). For this study the registry provided data on all first LT performed in patients with PBC between 1990 and 2021, with follow-up until March 16th 2021. Eligibility criteria encompassed; adults (≥18 years) undergoing donation after brain death (DBD) liver transplant (single organ) solely for PBC. Patients were excluded in case of inadequate or missing dates of follow-up or LT, or missing data on immunosuppressive therapies. This research was conducted in accordance with the principles of the Declaration of Helsinki and Istanbul. The ELTR adheres to GDPR. Each participating centre in the ELTR is responsible for obtaining informed consent from patients prior to registration, thereby the requirement for additional written consent for the ELTR is waived. All data provided by ELTR were anonymized, quality of data is ensured by randomly performed audits of contributing centres.

### Data and endpoints

ELTR data used for the current study included recipient and donor age, recipient and donor sex, type of donor (DBD grafts, donation after circulatory death grafts, living donor donation, domino procedure), total ischemia time, date of LT, centre where LT was performed, and usage of initial and maintenance immunosuppressive drugs. The type of CNI was primarily based on the last registered maintenance regimen (beyond the first month after LT). In case data on the maintenance immunosuppressive treatments was missing, the type of CNI was based on the registered immunosuppressive treatments during the initial phase (first month). In case there was no CNI registered among the immunosuppressive drugs or in case both CNIs were registered among the maintenance immunosuppressive treatments, patients were excluded from the analyses. The non-CNI immunosuppressive drugs were based on the registered maintenance regimen. Data on cirrhosis-related complications and laboratory parameters were not considered because of the nature of our study and the high rate of missing values among these variables in the ELTR. The clinical endpoints which were assessed in this study were graft loss and death, both irrespective of cause. Patients were considered to have lost their graft in case of a second LT or death.

### Statistical analyses

Categorical variables were expressed as counts and percentages (n, %) and continuous variables as medians and interquartile ranges. Follow-up time was calculated from date of first transplant to either graft loss or death. Patients who were alive without an event were censored at the last follow-up date as registered in the ELTR. Graft survival and overall patient survival were evaluated according to the type of CNI using the life-table Kaplan Meier method. Comparisons between both groups were made by log-rank test. For the primary analyses, patients with a follow-up duration <365 days (due to early loss to follow-up, a LT <365 days prior to data transfer, or early events post LT) were excluded from the survival analyses. Cox proportional hazard analyses were used to assess which factors were associated with graft or patient survival. When appropriate, polynomial terms were added to the model to account for non-linearity. The final model (model 1) was based on inclusion of variables with a *p* value of less than 0.1 in univariate analyses, with specific covariates retained as essential factors in the model (recipient sex, recipient age, donor age and year of LT as continuous variables). Besides, we performed a backward model selection procedure to assess additional variables for inclusion, ensuring a comprehensive examination of potential (additional) confounders. Data are presented as hazard ratio (HR) or adjusted HR (aHR) with 95% CIs. Several sensitivity Cox proportional hazard analyses were performed. First, analyses were repeated in various patient subgroups, including females, different age and calendar time categories, high volume centres (contributing over 50 LT in the dataset), and in those patients who had the same type of CNI registered in both their initial and their maintenance regimen. Second, the model estimates were assessed in analyses in which patients were included in case they had at least 90 days of event-free follow-up, rather than 365 days. Third, to increase the number of patients in the analyses, the associations between type of CNI and clinical outcomes were evaluated in a model which did not adjust for the use of other immunosuppressive drugs (MMF and steroids). Statistical analyses were performed with IBM SPSS Statistics, version 28.0.1.0 (142), software (Chicago, IL).

## Results

### Study population

**Fig. 1:**
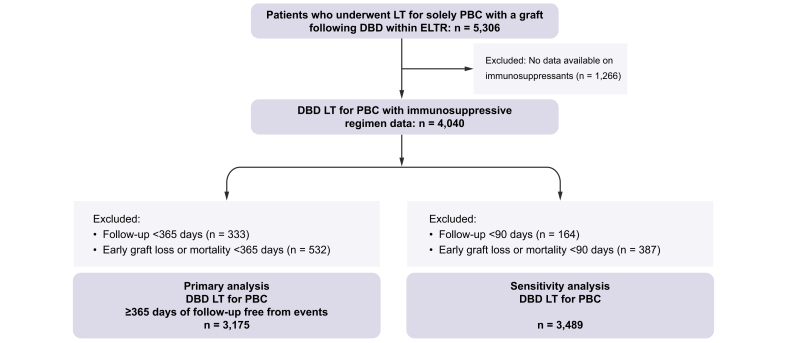
Study flowchart. The flowchart starts with 5,306 adults transplanted for PBC with a graft following DBD LT. Patients without data on immunosuppressants post LT were excluded. Data on immunosuppressive regimen was available in 4,040 patients, of whom 3,175 had at least 365 days of event-free follow-up and could be included for the primary analyses. DBD, donation after brain death donor; LT, liver transplant(ation); PBC, primary biliary cholangitis.

In primary analyses, 3,175 patients with at least 1 year of event-free follow-up after DBD LT for PBC were included as they had available data on the use of immunosuppressive drugs (Fig. 1). The baseline characteristics are shown in Table 1. Overall, 2,764 (87.1%) of the recipients were female and the median age at LT was 55.4 years (IQR 48.8–61.4). Recipients were followed for a median of 11.4 years (IQR 5.9–17.9), during which 120 patients underwent a second LT and 1,075 patients died. Tables S1 and S2 present the registered reasons for graft loss and death among those patients for whom these data were available within the dataset of the ELTR. For those still in follow-up after the first year, the overall 10-year graft survival was 80.9% (95% CI 79.3–82.5) and the overall patient survival was 82.9% (95% CI 81.5–84.3).

**Table 1:** Cohort characteristics.

	DBD cohort >1-year follow-up (N = 3,175)	Tac (n = 2,056)	CsA (n = 819)	*p* value
Median calendar year of LT	2001 (1996–2009)	2004 (1998–2011)	1996 (1994–2000)	<0.001
Median follow-up (years)	11.4 (5.9–17.8)	9.9 (4.7–16.5)	14.0 (8.3–19.7)	<0.001
Recipient age at LT	55.4 (48.8–61.4)	55.2 (48.5 – 61.4)	55.5 (48.8–61.0)	0.157
Recipient sex				0.005
Male	410/3,174 (12.9%)	279/2,055 (13.6%)	80/819 (9.8%)	
Female	2,764/3,174 (87.1%)	1,776/2,055 (86.4%)	739/819 (90.2%)	
Donor sex				0.278
Male	1,385/3,148 (44.0%)	880/2,041 (43.1%)	366/807 (45.4%)	
Female	1,763/3,148 (56.0%)	1,161/2,041 (56.9%)	441/807 (54.6%)	
Sex mismatch (recipient/donor)	1,322/3,147 (42.0%)	842/2,040 (41.2%)	346/807 (42.9%)	0.435
Donor age	43.8 (27.8–55.5)	45.9 (29.4–56.9)	39.9 (24.2–51.2)	<0.001
Total ischemia time (hours)	9.5 (7.3–12.0)	9.0 (7.0–11.4)	10.0 (7.7–13.4)	<0.001
Type of graft				<0.001
Full size	2,705/2,920 (92.6%)	1,646/1,805 (91.2%)	776/815 (95.2%)	
Reduced	19/2,920 (0.7%)	7/1,805 (0.4%)	8/815 (1.0%)	
Split	196/2,920 (6.7%)	152/1,805 (8.4%)	31/815 (3.8%)	
Mycophenolate maintenance	754/2,473 (30.5%)	619/1,818 (34.0%)	135/655 (20.7%)	<0.001
Steroids maintenance	763/2,473 (30.9%)	477/1,818 (26.2%)	286/655 (43.7%)	<0.001
Azathioprine maintenance	415/2,473 (16.8%)	211/1,818 (11.6%)	204/655 (31.1%)	<0.001

CsA, cyclosporin; DBD, donation after brain death; LT, liver transplant(ation); Tac, tacrolimus.

Patient characteristics for the overall cohort and separately for the Tac and CsA group. Continuous variables are shown as median and interquartile range and categorical variables are expressed as counts and percentages (n, %).

### Use of maintenance CNI and non-CNI drugs

**Fig. 2:**
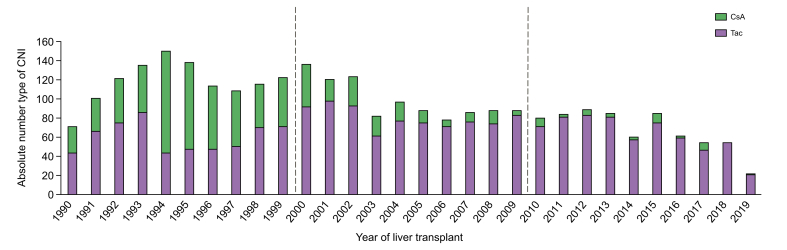
Maintenance calcineurin inhibitor by transplant year. Absolute numbers of patients in our primary analysis who either used Tac or CsA according to the year of LT. Type of CNI was primarily based on maintenance regimen, or if maintenance data was missing on the initial type of CNI. CNI, calcineurin inhibitor; CsA, cyclosporin; LT, liver transplant; Tac, tacrolimus.

Among the 3,175 patients, Tac was used by 2,056 (64.8%) patients and CsA by 819 (25.8%) patients, while 283 (8.9%) patients had no documented CNI and 17 (0.5%) patients had both types of CNI registered among their maintenance regimen. The type of CNI was based on maintenance CNI in 1,818 (88.4%) patients for Tac and in 655 (80.0%) patients for CsA. The initial and maintenance type of CNI differed in 221 (7.0%) patients; 53 (1.7%) patients switched from Tac to CsA and 168 (5.3%) patients switched from CsA to Tac. Fig. 2 shows the absolute number of patients in our analysis who either used Tac or CsA according to the year of LT. Among patients who were transplanted from 2010 onwards the percentage of patients on Tac remained above 85%. In concordance, the baseline variables differed between patients on Tac and those on CsA, with higher ischemia time in patients on CsA as opposed to those on Tac (10.0 [IQR 7.7–13.4] *vs.* 9.0 [IQR 7.0–11.4] hours, respectively, *p* <0.001) but older donor graft in patients on Tac as opposed to those on CsA (45.9 [29.4–56.9] *vs.* (39.9 [24.2–51.2], respectively, *p* <0.001) (Table 1).

Data on non-CNI maintenance immunosuppressive drugs were available in 2,473 (86.1%) patients with a single type of CNI. Of the 1,818 Tac users, 722 (39.7%) received Tac monotherapy, while 619 (30.1%), 477 (23.2%) and/or 211 (10.3%) patients received Tac alongside maintenance use of mycophenolate mofetil (MMF), steroids and/or azathioprine (AZA), respectively. Monotherapy of CsA was reported in 178 (27.2%) patients, while 286 (34.9%), 204 (24.9%), and 135 (16.5%) patients received CsA alongside maintenance use of steroids, AZA, and MMF, respectively.

### Immunosuppressive drugs in relation to clinical outcome

**Fig. 3:**
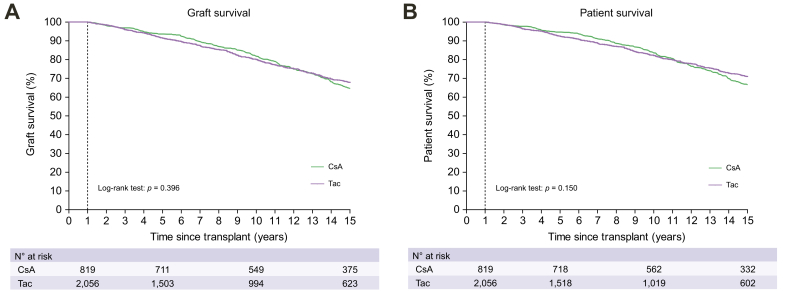
Graft and overall survival according to type of calcineurin inhibitor. Only patients with at least 1 year of follow-up were included in these Kaplan Meier survival curves for (A) graft survival (*p* = 0.396∗) and (B) patient survival (*p* = 0.150∗) according to CsA or Tac. ∗Survival was compared by log-rank test. CsA, cyclosporin; Tac, tacrolimus.

Among patients who used a single CNI, Tac was not associated with statistically significantly higher risk of graft loss (HR 0.946, 95% CI 0.833-1.075, *p =* 0.397) or death (HR 0.909, 95% CI 0.797-1.035, *p =* 0.150). In patients with at least 1-year of follow-up, the 10-year graft and patient survival were 80.0% (95% CI 78.0-82.0) and 82.2% (95% CI 80.2-84.2) for those on Tac *vs*. 81.8% (95% CI 79.1-84.5) and 83.6% (95% CI 80.9-86.3) for those on CsA (*p* >0.150 for both, Fig. 3). Registered maintenance use of MMF was associated with a lower long-term risk of graft loss (HR 0.768, 95% CI 0.643-0.917, *p =* 0.003) or death (HR 0.755, 95% CI 0.628-0.908, *p =* 0.003), while patients for whom steroids were registered among their maintenance immunosuppressive regimen had a higher risk of graft loss (HR 1.225, 95% CI 1.064-1.411, *p =* 0.005) or death (HR 1.243, 95% CI 1.075-1.436, *p =* 0.003). Use of AZA during follow-up was not associated with long-term outcome in univariate analyses for graft survival (HR 0.973, 95% CI 0.822-1.150) or patient survival (HR 0.988, 95% CI 0.832-1.172), *p* >0.745 for both, Table 2).

**Table 2:** Cox proportional hazards regression models for graft and patient survival.

	Univariate		Multivariable	
	HR (95% CI)	*p* value	aHR (95% CI)	*p* value
**Graft survival**				
Female (ref. male)	0.859 (0.725–1.018)	0.080	0.900 (0.734–1.104)	0.312
Recipient age at LT	1.034 (1.026–1.041)	<0.001	0.877 (0.820–0.938)	<0.001
Recipient age at LT^2^∗	1.002 (1.001–1.002)	<0.001	1.002 (1.001–1.002)	<0.001
Donor age	1.012 (1.008–1.016)	<0.001	1.008 (1.003–1.012)	<0.001
Total ischemia time (hours)	1.003 (0.984–1.023)	0.749	–	
Sex mismatch (R/D)	0.994 (0.884–1.117)	0.914	–	
Calendar year of LT	1.013 (1.001–1.024)	0.028	1.003 (0.988–1.018)	0.702
Tac use (ref. CsA)†	0.946 (0.833–1.075)	0.397	1.069 (0.915–1.249)	0.402
MMF†	0.768 (0.643–0.917)	0.003	0.718 (0.595–0.867)	<0.001
AZA†	0.973 (0.822–1.150)	0.745	–	
Steroids†	1.225 (1.064–1.411)	0.005	1.308 (1.126–1.519)	<0.001
**Patient survival**				
Female (ref. male)	0.846 (0.711–1.007)	0.061	0.888 (0.720–1.094)	0.264
Recipient age at LT	1.047 (1.039–1.055)	<0.001	0.894 (0.830–0.963)	0.003
Recipient age at LT^2^∗	1.002 (1.001–1.002)	<0.001	1.002 (1.001–1.002)	<0.001
Donor age	1.011 (1.007–1.015)	<0.001	1.007 (1.003–1.012)	0.002
Total ischemia time (hours)	1.004 (0.984–1.025)	0.670	–	
Sex mismatch (R/D)	0.997 (0.884–1.125)	0.967	–	
Calendar year of LT	1.007 (0.995–1.019)	0.229	0.997 (0.981–1.013)	0.687
Tac (ref. CsA)†	0.909 (0.797–1.035)	0.150	1.060 (0.904–1.243)	0.473
MMF†	0.755 (0.628–0.908)	0.003	0.716 (0.588–0.872)	<0.001
AZA†	0.988 (0.832–1.172)	0.887	–	
Steroids†	1.243 (1.075–1.436)	0.003	1.339 (1.148–1.562)	<0.001

AZA, azathioprine; CNI, calcineurin inhibitor; CsA, cyclosporine; (a)HR, (adjusted) hazard ratio; LT, liver transplant(ation); MMF, mycophenolate mofetil; Tac, tacrolimus.

For the primary analyses, patients with a follow-up duration <365 days due to early loss to follow-up, a LT <365 days prior to data transfer, or early events post LT, were excluded from the survival analyses.

∗ Polynomial term for age was added to the model to account for non-linearity and is consistently used in conjunction with the linear term of age.

† Maintenance use of immunosuppressant.

Adjusting for recipient age, recipient sex, donor age and year of LT did not alter the lack of association between the type of CNI and graft loss (aHR 1.069, 95% CI 0.915-1.249, *p =* 0.402) or overall death (aHR 1.060, 95% CI 0.904-1.243, *p =* 0.473) (Table 2). In this multivariable model, maintenance MMF remained associated with a lower risk of graft loss (aHR 0.718, 95% CI 0.595-0.867, *p <*0.001) and death (aHR 0.716, 95% CI 0.588-0.872, *p <*0.001), while maintenance steroid use remained associated with a higher risk of graft loss (aHR 1.308, 95% CI 1.126-1.519, *p <*0.001) or death (aHR 1.339, 95% CI 1.148-1.562, *p <*0.001). Table 3 describes the results of several sensitivity analyses. These analyses showed consistent aHR estimates for type of CNI, MMF and steroids with respect to graft loss or patient death in various subgroups of patients, as well as for follow-up starting 90 days as opposed to 365 days after LT. Additionally, similar results were obtained with respect to the association between type of CNI and clinical outcomes in an alternative Cox model which did not include MMF or steroids as covariates in order to maximize the number of patients in the analyses.

**Table 3:** Sensitivity analysis multivariate Cox proportional hazard models for graft and patient survival.

		Tac (ref. CsA)		MMF (ref. no MMF)		Steroids (ref. no steroids)	
	(n)	aHR (95% CI)	*p* value	aHR (95% CI)	*p* value	aHR (95% CI)	*p* value
**Graft survival**							
Females	2,083	1.081 (0.916–1.276)	0.356	0.681 (0.553–0.838)	<0.001	1.321 (1.125–1.551)	<0.001
Age <55.4#	1,191	1.272 (0.987–1.640)	0.063	0.686 (0.504–0.934)	0.017	1.309 (1.039–1.650)	0.022
Age ≥55.4#	1,193	0.969 (0.794–1.183)	0.758	0.754 (0.593–0.959)	0.022	1.321 (1.084–1.611)	0.006
Year of LT 1990–2000	983	1.115 (0.929–1.338)	0.224	0.803 (0.608–1.061)	0.123	1.256 (1.035–1.523)	0.021
Year of LT ≥2000	1,413	0.935 (0.670–1.303)	0.690	0.649 (0.503–0.838)	0.004	1.437 (1.126–1.835)	0.004
Initial = maintenance CNI‡	1,585	1.003 (0.787–1.277)	0.983	0.679 (0.542–0.851)	<0.001	1.343 (1.104–1.634)	0.003
High volume centres§	1,825	1.105 (0.930–1.312)	0.257	0.737 (0.591–0.918)	0.007	1.242 (1.056–1.461)	0.009
Alternative start of follow-up							
≥90 days post LT†	2,563	1.113 (0.958–1.294)	0.162	0.692 (0.578–0.828)	<0.001	1.251 (1.083–1.444)	0.002
Not adjusted for MMF or steroids							
Overall	2,782	0.931 (0.812–1.067)	0.304	–	–	–	–
**Patient survival**							
Females	2,083	1.069 (0.902–1.267)	0.441	0.679 (0.547–0.844)	<0.001	1.354 (1.148–1.598)	<0.001
Age <55.4#	1,191	1.275 (0.976–1.667)	0.075	0.682 (0.485–0.960)	0.028	1.400 (1.093–1.793)	0.008
Age ≥55.4#	1,193	0.969 (0.793–1.183)	0.754	0.745 (0.585–0.950)	0.017	1.317 (1.080–1.607)	0.007
Year of LT 1990–2000	983	1.112 (0.924–1.338)	0.263	0.818 (0.616–1.084)	0.162	1.257 (1.034–1.529)	0.022
Year of LT ≥2000	1,413	0.861 (0.612–1.213)	0.393	0.627 (0.478–0.824)	<0.001	1.563 (1.209–2.021)	<0.001
Initial = maintenance CNI‡	1,585	0.986 (0.770–1.264)	0.914	0.685 (0.542–0.867)	0.002	1.372 (1.120–1.680)	0.002
High volume centres§	1,828	1.100 (0.923–1.311)	0.289	0.733 (0.582–0.922)	0.008	1.265 (1.070–1.494)	0.006
Alternative start of follow-up							
≥90 days post LT†	2,563	1.087 (0.931–1.268)	0.290	0.697 (0.577–0.842)	<0.001	1.300 (1.120–1.508)	<0.001
Not adjusted for MMF or steroids							
Overall	2,782	0.913 (0.794–1.050)	0.201	–	–	–	–

CNI, calcineurin inhibitor; CsA, cyclosporine; (a)HR, (adjusted) hazard ratio; LT, liver transplant(ation); MMF, mycophenolate mofetil; Tac, tacrolimus.

Sensitivity analyses of primary model are presented. The full model includes: recipient sex, recipient age, recipient age^2^, donor age, and year of transplant, type of calcineurin inhibitor, use of maintenance mycophenolate and steroids. The aHRs with 95% CIs are presented, with values below 1.0 favouring Tac, values below 1.0 favouring use of MMF, and values below 1.0 favouring steroids.

# Cut-off based on median recipient age.

‡ Subgroup of patients who had the same type of CNI registered among their initial and maintenance immunosuppressive regimen.

§ Centres with over 50 PBC transplants within the European Liver Transplant Registry.

† Analyses in which patients were included from 90 days post LT, rather than from 365 days. Sensitivity analysis not including MMF and steroid use in the model to assess the estimates for type of CNI in the overall study population.

## Discussion

In this largest cohort study to date, no difference was observed in long-term graft or patient survival for patients with PBC who used Tac or CsA following DBD liver transplantation. Further, use of MMF was associated with a reduced risk of graft loss or death while maintenance steroid use was associated with worse outcomes. These findings were robust following multivariable adjustment as well as in a variety of clinically relevant sensitivity analyses. The absent association between the type of CNI and long-term post-LT outcomes is relevant considering the ongoing discussion on the negative impact of Tac on rPBC. For this study, the power of the ELTR dataset enabled us to assess the type of CNI in relation to graft and patient survival as more solid clinical endpoints. Our study therefore supports the preferred use of Tac in the population with PBC, which is in general the first-choice CNI following LT. While we observed a substantial increase in use of Tac following LT among patients with PBC in Europe over recent years, there remain centres where CsA replaces Tac following LT in patients with PBC. In addition, the results suggest that there may be a benefit of combining Tac with MMF to spare the side effects of higher dosed Tac alone.

So far, studies assessing the differences in clinical outcome according to the type of CNI following LT in patients with PBC primarily focused on rPBC as an endpoint. Higher recurrence rates and shorter time to recurrence were initially observed in small studies conducted in the 1990s and early 2000s.[20], [21], [22], [23] More recently, non-randomized studies have described a higher risk of rPBC with Tac following LT with HRs ranging from 2.0 to 3.4.^17^^,^^18^ However, these results were not consistent, as a Japanese multicentre living donor study found a completely opposite increased risk of rPBC with CsA (aHR 2.5) *vs.* Tac.^24^ Despite prior believes, rPBC was recently associated with unfavourable graft and patient survival in the study by Montano-Loza *et al.*, performed within the selected international centres of the Global PBC Study Group. Still, the endpoint of rPBC has limitations as it remains a histological diagnosis. Histopathological assessment based on different features may lead to varying recurrence rates.^25^ Results may also be influenced by differences in post-LT programs, including either protocol liver biopsies or clinically indicated biopsies. In addition, a strong rationale to support an increased risk of rPBC with Tac over CsA is lacking. One of the described hypotheses, from a small genome-wide sequencing study, is related to a potential risk loci (IL12) for susceptibility to rPBC as Tac and CsA have different mechanisms for the inhibition of IL-2, which in turn may affect IL-12.^26^ Both these cytokines are involved in the regulation of T cells, and the development of autoreactive T-helper 1 cells has been associated with PBC development.^27^ However, it is unclear how these signalling pathways specifically influence PBC recurrence. Another conjecture posits that there is an increased potential for virological or environmental triggers with the use of Tac as a more potent immunosuppressant.^28^ Therefore, focusing solely on rPBC does not account for the potential benefits of Tac over CsA, for instance with respect to the more effective prevention of ACR and reduced 1-year and 3-year post-transplant mortality.^28^^,^^29^ It is thus relevant that the current study assessed the association between type of CNI and long-term graft and patient survival. Although the median time to rPBC is very heterogeneous in the published literature, one of the more recent and larger cohorts indicated a median time of 4.4 years.^17^ The earliest case with rPBC has even been described as early as 4 months after LT.^23^^,^^25^ In case there is a relevant relation between type of CNI and rPBC, we consider the ELTR to be a valid cohort to assess this in relation to long-term graft and patient survival. The median follow-up of 11.4 years presented here aligns closely with that of the study wherein rPBC was associated with an unfavourable clinical outcome.^18^

Our study does not exclude the possibility that Tac indeed increases the incidence of rPBC, as this outcome measure is not generally available in the ELTR dataset. Nevertheless, the results on solid clinical endpoints as presented here are reassuring. The rates of rPBC as a reason for graft loss or patient mortality were similar for the patient on maintenance Tac (20.0% for graft loss and 4.7% for patient death) and CsA (20.7% graft loss and 3.0% patient death). Still, these results should be interpreted cautiously as the available data were limited and there was no uniform standardised protocol to assess rPBC across the European LT centres. Even though there was no difference in graft and patient survival between those on Tac or CsA in the study presented here, there are several arguments to favour Tac as the preferred CNI after LT for PBC. First, preventive use of UDCA after LT for PBC was recently shown to reduce the risk of rPBC and improve survival, while this was not standard medical management during the time-period of the cohort studies indicating an increased rPBC rate with Tac.^18^^,^^30^ Standard UDCA use after LT for PBC today may thus mitigate the potentially increased rPBC risk of Tac. Second, it is relevant to consider that the treatment options for PBC have extended with the development of the multiple nuclear receptor agonists. Although solid data on the use of these second-line add-on drugs in the setting of rPBC have yet to be presented, it may be anticipated that these drugs would be effective based on their working mechanism.^31^ Third, patients with immune-mediated liver diseases are known to be at increased risk of rejection,^32^^,^^33^ which has a well-documented negative impact on post-LT outcomes. The ELTR dataset is lacking details on possible rejection over time, so the association between type of CNI and ACR could not be assessed. Importantly, however, randomized-controlled trials have clearly shown that Tac is the preferred CNI to prevent rejection.^34^ Prioritizing the prevention of rejection should likely take precedence over the possible prevention of rPBC. Apart from the direct liver-related consequences of hepatic inflammatory activity, there is an indirect negative impact on patient outcomes as a result of the required intensification of immunosuppressive drugs in case of rejection. Our finding of a 1.3-fold higher risk of graft loss and death with maintenance steroids aligns with this. However, residual confounding and indication bias may be specifically relevant in relation to this finding and a causal relation can thus not be concluded.

Interestingly, we observed an almost 30% lower risk of graft loss or death in patients who used MMF in combination with their CNI (aHR 0.72, *p <*0.001 for both). The use of the antimetabolite MMF has consistently increased over the past two decades, among other reasons, to reduce the CNI dosage and thereby reduce CNI side effects. Indeed, patients on CNIs have an increased risk of nephrotoxicity, diabetes mellitus, hypertension, and *de novo* malignancies.[35], [36], [37], [38] The EASL guidelines discourage complete CNI withdrawal, however, due to significantly increased risk of ACR. Reducing the CNI dose can be effectively done with the introduction of MMF, and this strategy improved graft rejection rates and can limit the long-term disadvantageous effects of CNIs.[39], [40], [41], [42], [43], [44] Still, MMF may be limited by side effects as well, which include nausea, vomiting, diarrhoea, abdominal pain and a possibly higher risk of opportunistic and viral infections.[45], [46], [47] The potential long-term benefit on solid clinical endpoints of combination therapy, as shown in our study, may not be limited to the population with PBC. Indeed, similar beneficial results for MMF have recently been described for graft loss in a post-LT study among patients with primary sclerosing cholangitis (HR 0.82).^48^ Although randomized-controlled data are lacking, our results argue that the threshold to institute the combination of Tac and MMF should be low.

Notwithstanding the size and nature of the ELTR dataset, containing data for practically every LT centre in Europe, some limitations should be acknowledged. As the ELTR dataset has not been designed to assess the relation between type of CNI and clinical outcome, details on rPBC and ACR episodes are lacking. Also, there was no data on the use of UDCA or the dosages and duration of maintenance immunosuppressive drugs over time. For this reason, we conducted a sensitivity analysis in which patients were only included who had the same type of CNI registered among their initial and maintenance regimen. Over the last decades there have been major improvements in post-LT outcomes, especially related to an improved 1-year graft and patient survival.^14^^,^^49^ Considering our primary focus on the long-term impact of the type of CNI, we excluded early graft loss or death during the first year. These events may be largely related to causes such as surgical failure, primary dysfunction and early infections. However, as ACR may occur early, we have performed a sensitivity analysis in which follow-up started at 90 days after LT instead of at 1 year after LT. In this analysis, the results regarding the association between the immunosuppressive drugs and graft and patient survival were similar.

In conclusion, in this large cohort of patients with PBC who underwent LT, there was no difference in long-term graft or patient survival according to the type of CNI. The results of our study should thus reassure transplant hepatologists to continue the use of Tac after LT in the population with PBC. Use of maintenance steroids following LT identified a subgroup of patients with an unfavourable clinical outcome. In contrast, concomitant use of MMF was associated with a substantially improved graft and patient survival. While further studies should assess the clinical impact of combination treatment with Tac and MMF post LT as well, our results provide support for adding MMF to Tac in order to reduce CNI-related side effects.

## Abbreviations

ACR, acute cellular rejection; aHR, adjusted hazard ratio; AZA, azathioprine; CNI, calcineurin inhibitor; CsA, cyclosporin; DBD, donation after brain death; ELTR, European Liver Transplant Registry; HR, hazard ratio; MMF, mycophenolate mofetil; LT, liver transplant(ation); PBC, primary biliary cholangitis; rPBC, recurrence of primary biliary cholangitis; Tac, tacrolimus; UDCA, ursodeoxycholic acid.

## Financial support

Christophe Corpechot received fees for consulting work from 10.13039/100018262Intercept Pharmaceuticals, Ipsen, Cymabay Therapeutics, GlaxoSmithKline, and Calliditas Therapeutics, and received unrestricted grants from Arrow Génériques and Intercept Pharmaceutical France. Michael Heneghan reports financial research support by The King’s College Hospital Charity. Caroline den Hoed received speakers fees from Orphalan ltd, Chiesi, unrestricted grants from Sobi, and received fees for consulting work from Takeda Pharma, Abacus medicine and Astellas. The institution of Adriaan J. van der Meer received speakers fees from Zambon Nederland B.V. and AOP Health, received unrestricted grants from CymaBay Therapeutics, Gilead Sciences, MSD and Zambon Nederland B.V., and received fees for consulting work from Intercept Pharma, AOP Health, CymaBay Therapeutics, and Ipsen.

## Conflict of interest

All authors declare that they have no conflict of interest regarding the content of this manuscript.

Please refer to the accompanying ICMJE disclosure forms for further details.

## Authors’ contributions

Study concept and design: MH, HM, WP, CDH, AM. Data acquisition: VK, RA, PT, WP, HP, SDM, DM, MH, PL, GO, DT, MA, HM, CDH, MH, AM. Data Analysis: MH, AM. Data Interpretation: AM, SDM, RV, HM, MH. Drafting manuscript MH, AM. Critical revision for important intellectual content and final approval: all authors.

## Data availability statement

The data is available upon request for specific research questions from the ELTR registry, following the rules presented on the website of the registry.
