# Supplementary material for: Type of calcineurin inhibitor and long-term outcomes following liver transplantation in patients with primary biliary cholangitis – an ELTR study
Source: JHEP Rep. 2024 Apr 25;6(8):101100. doi: 10.1016/j.jhepr.2024.101100 (PMC11263784; doi:10.1016/j.jhepr.2024.101100)
Supplement: Multimedia component 1 [file mmc1.pdf]

Association between type of calcineurin inhibitor and long-term outcomes following liver transplantation in patients with primary biliary cholangitis – an ELTR study

Maria C. van Hooff, Rozanne C. de Veer, Vincent Karam, Rene Adam, Pavel Taimr, Wojciech G. Polak, H. Pashtoun, Sarwa Darwish Murad, Christophe Corpechot, Darius Mirza, Michael Heneghan, Peter Lodge, Gabriel C. Oniscu, Douglas Thorburn, Michael Allison, Herold J. Metselaar, Caroline M. den Hoed, Adriaan J. van der Meer, for the European Liver and Intestine Transplant Association (ELITA)

Table of contents

Table S1……………………………………………………………………..………………. 2

Table S2... 2

Table S1. Causes of graft loss after the first year following liver transplantation according to type of CNI

	Tac n, (%)	CsA n, (%)	Non CNI/Both CNI n, (%)
	n=80	n=29	n=11
**Infection**	2 (2.5)	2 (6.9)	0 (0.0)
**Liver complications**			
Chronic rejection	10 (12.5)	3 (10.3)	4 (36.5)
Arterial thrombosis	2 (2.5)	1 (3.4)	0 (0.0)
Biliary Anastomotic	3 (3.8)	1 (3.4)	1 (9.1)
Biliary non-anastomotic	2 (2.5)	2 (6.9)	2 (18.2)
Other liver complication	2 (2.5)	1 (3.4)	0 (0.0)
**Recurrence PBC**	16 (20.0)	6 (20.7)	0 (0.0)
**Gastrointestinal complications**	1 (1.3)	0 (0.0)	0 (0.0)
**Other**	0 (0.0)	2 (6.9)	0 (0.0)
**Not available**	42 (52.5)	11 (37.9)	4 (36.4)

Abbreviations : CNI, calcineurin inhibitor; CsA, cyclosporine; IS, immunosuppressive; Tac, Tacrolimus

Table S2. Causes of death after the first year following liver transplantation according to type of CNI

	Tac n, (%)	CsA n,(%)	Non CNI/ Both CNI n,(%)
	n=592	n=367	n=116
**Infection**	120 (20.3)	61 (16.6)	23 (19.8)
**Neoplasia**	90 (15.2)	51 (13.9)	14 (12.1)
**Bone marrow depression**	1 (0.2)	0 (0.0)	0 (0.0)
**Liver complications**			
Acute rejection	1(0.2)	0 (0.0)	0 (0.0)
Chronic rejection	11 (1.9)	8 (2.2)	2 (1.7)
Arterial thrombosis	3 (0.5)	2 (0.5)	0 (0.0)
Hepatic vein thrombosis	1 (0.2)	0 (0.0)	0 (0.0)
Outflow impairment	0 (0.0)	1 (0.3)	0 (0.0)
De novo hepatitis B virus	0 (0.0)	1 (0.3)	0 (0.0)
Other liver complication	7 (1.2)	5(1.4)	3 (2.6)
**Biliary complications**	9 (1.5)	5 (1.4)	1 (0.9)
**Gastrointestinal complications**			
Gastrointestinal haemorrhage	8 (1.4)	1 (0.3)	1 (0.9)
Other Gastrointestinal complication	5 (0.8)	3 (0.8)	1 (0.9)
**Cardiovascular complications**	51 (8.6)	29 (7.9)	4 (3.4)
**Pulmonary Embolism**	3 (0.5)	1 (0.3)	3 (2.6)
**Cerebrovascular complications**	23(3.9)	20 (5.4)	7 (6.0)
**Kidney failure**	10 (1.7)	11 (3.0)	6 (5.2)
**Noncompliance IS therapy**	4 (0.7)	0 (0.0)	0 (0.0)
**Trauma**	2 (0.3)	1 (0.3)	0 (0.0)
**Other**	19 (3.4)	27 (7.4)	7 (6.0)
**Recurrence of disease**	28 (4.7)	11 (3.0)	5 (4.3)
**Not available**	196 (33.1)	129 (35.1)	39 (33.6)

Abbreviations : CNI, calcineurin inhibitor; CsA, cyclosporine; IS, immunosuppressive; Tac, Tacrolimus
